# Glutamatergic projections from homeostatic to hedonic brain nuclei regulate intake of highly palatable food

**DOI:** 10.1038/s41598-020-78897-9

**Published:** 2020-12-16

**Authors:** Ashley E. Smith, Kehinde O. Ogunseye, Julia N. DeBenedictis, Joanna Peris, James M. Kasper, Jonathan D. Hommel

**Affiliations:** 1 Department of Neuroscience, Cell Biology and Anatomy, University of Texas Medical Branch, Galveston, TX USA; 2 Center for Addiction Research, University of Texas Medical Branch, Galveston, TX USA; 3 Institute for Translational Science, University of Texas Medical Branch, Galveston, TX USA; 4 Masters in Nutrition Program, University of Texas Medical Branch, Galveston, TX USA; 5 Department of Pharmacodynamics, College of Pharmacy, University of Florida, Gainesville, FL USA; 6 Department of Pharmacology and Toxicology, University of Texas Medical Branch, Galveston, TX USA

**Keywords:** Feeding behaviour, Neural circuits

## Abstract

Food intake is a complex behavior regulated by discrete brain nuclei that integrate homeostatic nutritional requirements with the hedonic properties of food. Homeostatic feeding (i.e. titration of caloric intake), is typically associated with hypothalamic brain nuclei, including the paraventricular nucleus of the hypothalamus (PVN). Hedonic feeding is driven, in part, by the reinforcing properties of highly palatable food (HPF), which is mediated by the nucleus accumbens (NAc). Dysregulation of homeostatic and hedonic brain nuclei can lead to pathological feeding behaviors, namely overconsumption of highly palatable food (HPF), that may drive obesity. Both homeostatic and hedonic mechanisms of food intake have been attributed to several brain regions, but the integration of homeostatic and hedonic signaling to drive food intake is less clear, therefore we aimed to identify the neuroanatomical, functional, and behavioral features of a novel PVN → NAc circuit. Using viral tracing techniques, we determined that PVN → NAc has origins in the parvocellular PVN, and that PVN → NAc neurons express VGLUT1, a marker of glutamatergic signaling. Next, we pharmacogenetically stimulated PVN → NAc neurons and quantified both gamma-aminobutyric acid (GABA) and glutamate release and phospho-cFos expression in the NAc and observed a robust and significant increase in extracellular glutamate and phospho-cFos expression. Finally, we pharmacogenetically stimulated PVN → NAc which decreased intake of highly palatable food, demonstrating that this glutamatergic circuitry regulates aspects of feeding.

## Introduction

Obesity is an alarming chronic health crisis that affects almost 40% of the adult population in the United States^1^. Obese individuals present a challenging public health problem due to increased risk for several life-threatening and costly co-morbidities including diabetes, metabolic syndrome, cardiovascular disease, and cancer^2^. Additionally, obesity is strongly linked to mental illness, including anxiety and depression, as well as neurodegenerative diseases that ultimately lead to cognitive decline^3,4^. Food intake is essential for life and is elicited through homeostatic demand and the hedonic value of food. One maladaptive feeding behavior that contributes to obesity is pathological overconsumption of highly palatable food (HPF). HPF is extremely reinforcing in both humans and rodents alike and is typically high in fat. Fat is perhaps one of the most palatable macronutrients due to its taste, texture, and caloric density- all of which drive pathological overconsumption^5–8^. The reinforcing properties of HPF are encoded by the nucleus accumbens (NAc), which is a well-known neural substrate of reinforcement and goal-directed behaviors that receives input from feeding-related brain regions^9,10^.

Food intake is also driven by metabolic energy demand, which is regulated by hypothalamic nuclei, specifically the paraventricular nucleus of the hypothalamus (PVN). The PVN integrates a multitude of central and peripheral signals to regulate food intake and energy balance. One manner in which the PVN may regulate reinforcement is through its direct inputs to the NAc^11,12^. PVN → NAc connectivity coordinates social reinforcement, but it is unclear whether the interplay of PVN → NAc regulates feeding behaviors even though both the PVN and the NAc have been implicated in the control of feeding behaviors^9,11,13–19^. However, the direct interplay of these regions, including functional mechanisms of PVN → NAc signaling, in regulating feeding behavior is unknown. Here, we identify PVN → NAc as a novel regulator of intake of highly palatable food (HPF). While the PVN is a link between homeostatic and hedonic brain nuclei, it is uncertain whether PVN inputs to the NAc might modulate hedonic feeding. Here, we define PVN → NAc as a novel source of glutamatergic input to the NAc and establish that PVN → NAc regulates intake of HPF in rats.

## Results

### PVN → NAc projection neurons originate from the parvocellular region of the PVN

**Figure 1:**
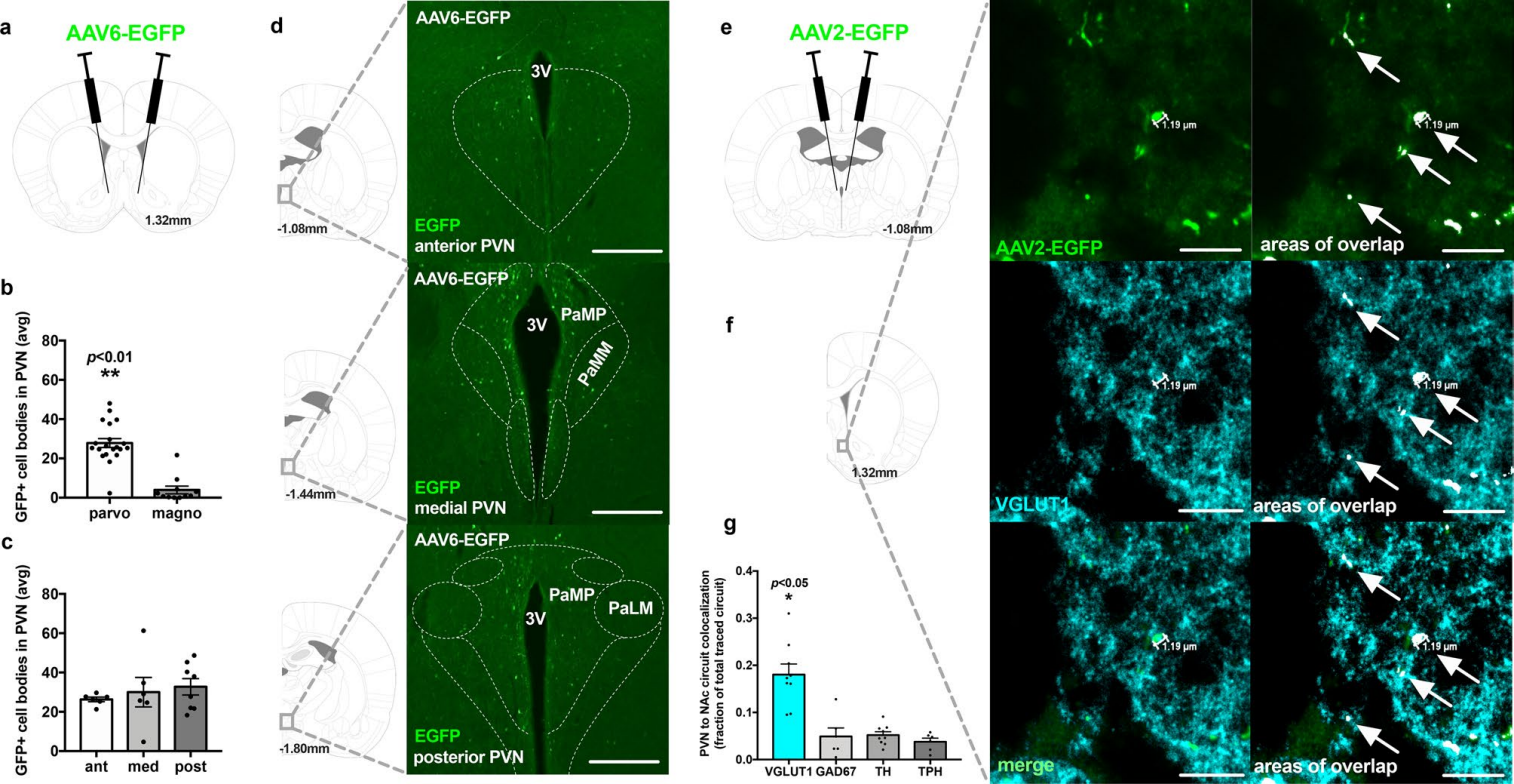
PVN → NAc projection neurons originate from parvocellular PVN and co-localize with VGLUT1. (**A**) viral targeting approach to label PVN → NAc cell bodies. (**B**) PVN cell bodies that project to the NAc are localized to parvocellular regions of the PVN. n = 3, **p < 0.01 by unpaired T-test. (**C**) PVN cell bodies that project to the NAc are uniformly localized in anterior, medial, and posterior PVN (n = 3, *p* > 0.05 by one-way ANOVA). (**D**) representative PVN images from n = 3 brains of anterior, medial, and posterior PVN with outlined structures used in quantification. (**E**) viral targeting approach to label PVN → NAc terminals. (**F**) PVN → NAc terminals strongly localize with VGLUT1 compared to GAD_67_, TH and TPH (n = 4, *p < 0.05 by one-way ANOVA). (**G**) representative NAc images from n = 4 brains illustrating PVN → NAc terminals overlap with VGLUT1. *3 V* third ventricle, *PaMP* paraventricular hypothalamic nucleus, medial parvicellular part, *PaMM* paraventricular hypothalamic nucleus, medial magnocellular part, *PaLM* paraventricular hypothalamic nucleus, lateral magnocellular part.

To define the origin of PVN inputs to the NAc, we administered a retrograde tracer to the NAc and examined green fluorescent protein (GFP)-labeled PVN cell bodies distributed throughout magnocellular and parvocellular regions of the PVN (Fig. 1A). Quantification of GFP-positive cell bodies revealed that PVN → NAc cell bodies are highly localized to parvocellular regions of the PVN (Fig. 1B; **p < 0.01), and are uniformly distributed throughout anterior, medial, and posterior regions of the PVN (Fig. 1C; *p* > 0.05, no significance). The presence of GFP-positive cell bodies throughout parvocellular regions of the PVN is evidence of a pathway arising in the PVN and projecting directly to the NAc (Fig. 1D). A critical functional distinction exists between parvocellular and magnocellular regions of the PVN- the parvocellular region drives intake of highly palatable food, whereas the magnocellular region did not^20^. The localization of PVN → NAc neurons to the parvocellular region provides neuroanatomical evidence that the PVN → NAc pathway may regulate feeding behavior.

### PVN → NAc projection neurons co-localize with VGLUT1

We also profiled PVN → NAc using an anterograde tracer in the PVN to identify co-localization of GFP-labeled terminals in the NAc with specific neurotransmitter markers (Fig. 1E). We performed immunohistochemistry on PVN → NAc terminals with VGLUT1 (vesicular glutamate transporter 1, glutamatergic neuron marker), GAD_67_ (glutamic acid decarboxylase 67, GABAergic neuron marker), TH (tyrosine hydroxylase, dopaminergic neuron marker), and TPH (tryptophan hydroxylase, serotoninergic neuron marker), and quantified co-localization events with each marker. Interestingly, we identified that PVN → NAc terminals co-localize most frequently with VGLUT1 (Fig. 1F,G; *p < 0.05), and representative images of PVN → NAc terminal staining with VGLUT1 reveal multiple co-localization events presynaptically in the NAc (Fig. 1F,G). This finding is consistent with the role of NAc glutamate in feeding behavior, and provides additional neuroanatomical evidence for glutamate as the major neurotransmitter involved in PVN → NAc signaling^9,13^.

### hM3d-induced stimulation of PVN → NAc increases extracellular glutamate in the NAc

**Figure 2:**
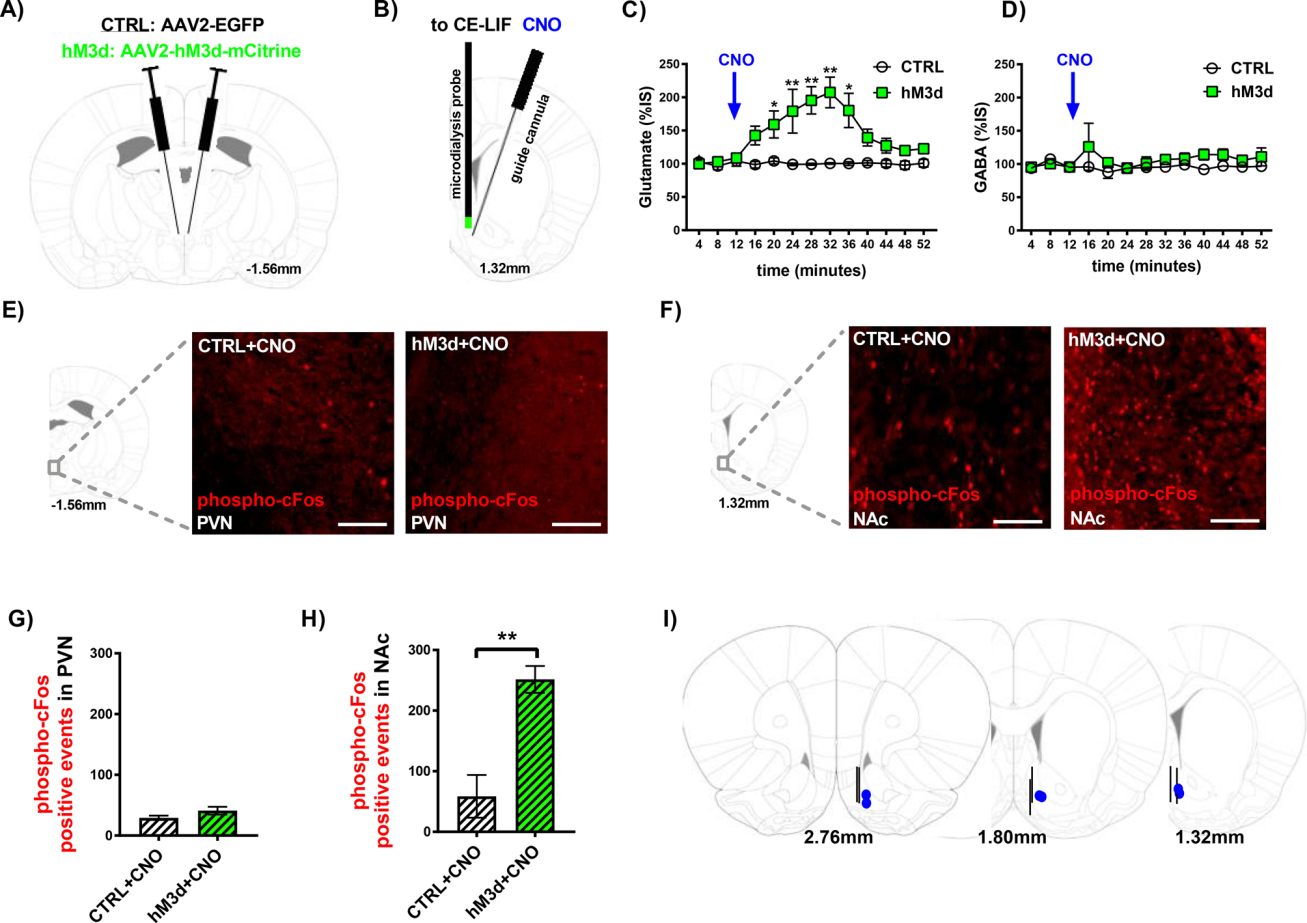
hM3d-induced stimulation of PVN → NAc increases extracellular glutamate and phospho-cFos expression in the NAc. (**A**) PVN CTRL and hM3d viral targeting approach. (**B**) NAc microdialysis probe placement to collect and quantify dialysate via CE-LIF methodology (left) and NAc guide cannula placement to administer CNO directly to NAc (right). (**C**) Pharmacogenetic stimulation of PVN → NAc neurons induced a robust, sustained increase in extracellular glutamate (CTRL n = 3, hM3d n = 3, *p < 0.05 and **p < 0.01 by 2-way ANOVA). (**D**) Pharmacogenetic stimulation of PVN → NAc neurons did not increase extracellular GABA (CTRL n = 3, hM3d n = 3, *p* > 0.05 by 2-way ANOVA). (**E**) Representative IHC images of phospho-cFos expression in the PVN of CTRL + CNO (left) and hM3d + CNO (right) rats show minimal staining. (**F**) Representative IHC images of phospho-cFos expression in the NAc of CTRL + CNO (left) show minimal staining. Representative IHC images of phospho-cFos expression in the NAc of hM3d + CNO (right) rats show increased staining. (**G**) Stimulation of PVN → NAc did not alter phospho-cFos expression in the PVN of CTRL + CNO vs. hM3d + CNO rats (CTRL n = 3, hM3d n = 3, *p* > 0.05 by unpaired T-test). (**H**) Stimulation of PVN → NAc did not alter phospho-cFos expression in the NAc of CTRL + CNO rats, but significantly increases phospho-cFos expression in the NAc of hM3d + CNO rats (CTRL n = 3, hM3d n = 3, **p < 0.01 by unpaired T-test). (**I**) Post-mortem placement of NAc guide cannula (blue dots) and NAc microdialysis probe (black lines). *CE-LIF* capillary electrophoresis with laser induced fluorescence, *CNO* clozapine-N-oxide, *IHC* immunohistochemistry.

To determine the functional mechanism of PVN → NAc signaling, we used a pharmacogenetic approach to stimulate neurotransmitter release from PVN → NAc projections. We used AAV2 to transduce PVN → NAc neurons with the excitatory designer receptor exclusively activated by a designer drug (DREADD) hM3d or an EGFP-expressing control vector (CTRL) into the PVN (Fig. 2A). This viral paradigm allows for the transport of hM3d to terminal regions of PVN efferents, including the NAc. To selectively stimulate the PVN → NAc pathway, clozapine-N-oxide (CNO) was micro-infused directly into the NAc to trigger presynaptic release of neurotransmitters (Fig. 2B). Accumbal GABA and glutamate were identified and quantified in real time via capillary electrophoresis followed by laser-induced fluorescence (Fig. 2B). Pharmacogenetic stimulation of PVN → NAc resulted in a robust, sustained increase in extracellular glutamate in hM3d rats compared to CTRL rats (Fig. 2C; *p < 0.05, **p < 0.01). This effect was observed immediately upon CNO administration for approximately 45 min (Fig. 2C). No change in extracellular GABA was observed in either hM3d or CTRL rats (Fig. 2D; *p* > 0.05, no significance), which supports that PVN → NAc predominately uses glutamate.

### Increased extracellular glutamate from PVN → NAc corresponds to increased phospho-cFos expression in the NAc of hM3d + CNO rats

Given that PVN → NAc release glutamate, we sought to identify the consequences of PVN → NAc stimulation on neuronal excitability both locally (PVN) and downstream (NAc). Therefore, we examined phosphorylated-cFos (phospho-cFos) protein expression in the PVN and the NAc of CTRL and hM3d rats following intra-NAc administration of CNO (CTRL + CNO, hM3d + CNO) (Fig. 2E-H). Representative IHC images show few phospho-cFos positive events in the PVN of CTRL + CNO and hM3d + CNO rats (Fig. 2E; p > 0.05, no significance). In the NAc, we observed few phospho-cFos positive events in CTRL + CNO rats (Fig. 2F; p < 0.05, no significance). However, in the NAc of hM3d + CNO rats, we observed several phospho-cFos positive events (Fig. 2F). Quantification of phospho-cFos positive events confirmed minimal phospho-cFos in the PVN of CTRL + CNO and hM3d + CNO rats (Fig. 2G; *p* > 0.05, no significance). Accordingly, phospho-cFos positive events were significantly elevated in the NAc of hM3d + CNO rats (Fig. 2H; **p < 0.01). Taken together, these data support the specificity of our approach- only presynaptic DREADDs are stimulated by intra-NAc CNO administration. Additionally, intra-NAc CNO did not alter the excitability of neurons locally- glutamate release from PVN → NAc increases the excitability of neurons downstream in the NAc. After euthanasia, we assessed placement of the microdialysis probe (black line) and guide cannula (blue) in the NAc for CTRL and hM3d rats and eliminated any misses from data analysis (Fig. 2I).

### hM3d-induced stimulation of PVN → NAc did not alter locomotor activity

**Figure 3:**
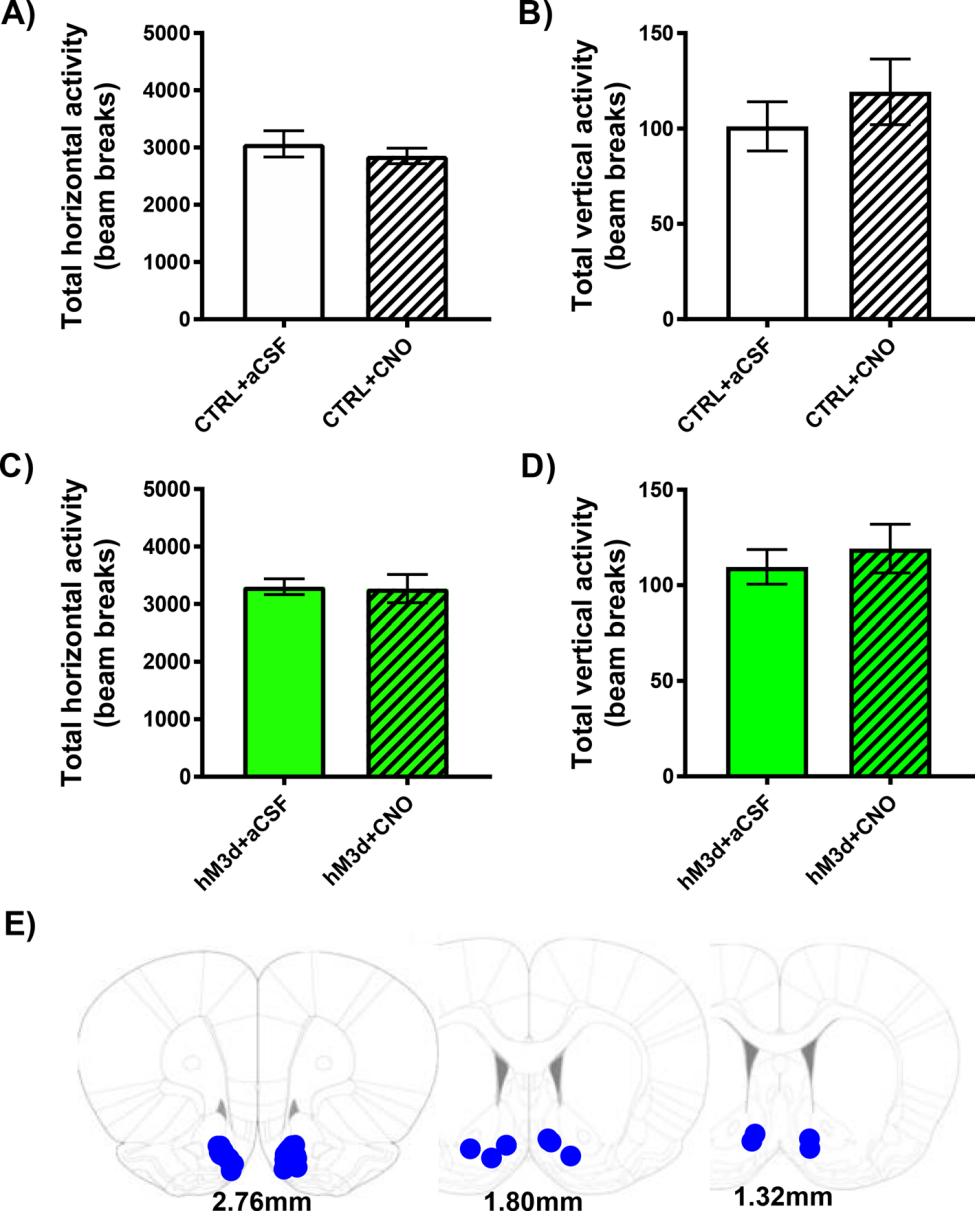
hM3d-induced stimulation of PVN → NAc did not alter locomotor activity. (**A**) Intra-NAc aCSF or CNO administration did not alter total horizontal activity in CTRL + aCSF or CTRL + CNO rats (n = 11, *p* > 0.05 by paired T-test). (**B**) Intra-NAc aCSF or CNO administration did not alter total vertical activity in CTRL + aCSF or CTRL + CNO rats (n = 11, *p* > 0.05 by paired T-test). (**C**) Intra-NAc aCSF or CNO administration did not alter total horizontal activity in hM3d + aCSF or hM3d + CNO rats (n = 15, *p* > 0.05 by paired T-test. (**D**) Intra-NAc aCSF or CNO administration did not alter total vertical activity in hM3d + aCSF or hM3d + CNO rats (n = 15, *p* > 0.05 by paired T-test). (**E**) Post-mortem placement of NAc guide cannula (blue dots).

Because increases in extracellular glutamate could manifest as increases in locomotor activity, we quantified total horizontal and total vertical locomotor activity in both CTRL and hM3d rats after intra-NAc administration of aCSF or CNO (Fig. 3). A paired T-test revealed no significant differences in total horizontal activity between CTRL + aCSF and CTRL + CNO rats (Fig. 3A; n = 11; *p* > 0.05, no significance), and no significant differences in total vertical activity between CTRL + aCSF and CTRL + CNO rats (Fig. 3B; *p* > 0.05, no significance). Similarly, we did not observe significant differences in total horizontal activity between hM3d + aCSF and hM3d + CNO rats (Fig. 3C; n = 15; *p* > 0.05, no significance), and no significant differences in total vertical activity between hM3d + aCSF and hM3d + CNO rats (Fig. 3D; n = 15; *p* > 0.05, no significance). Placement of NAc guide cannula used for aCSF or CNO micro-infusion was assessed after euthanasia (Fig. 3E).

### hM3d-induced stimulation of PVN → NAc decreases intake of HPF

**Figure 4:**
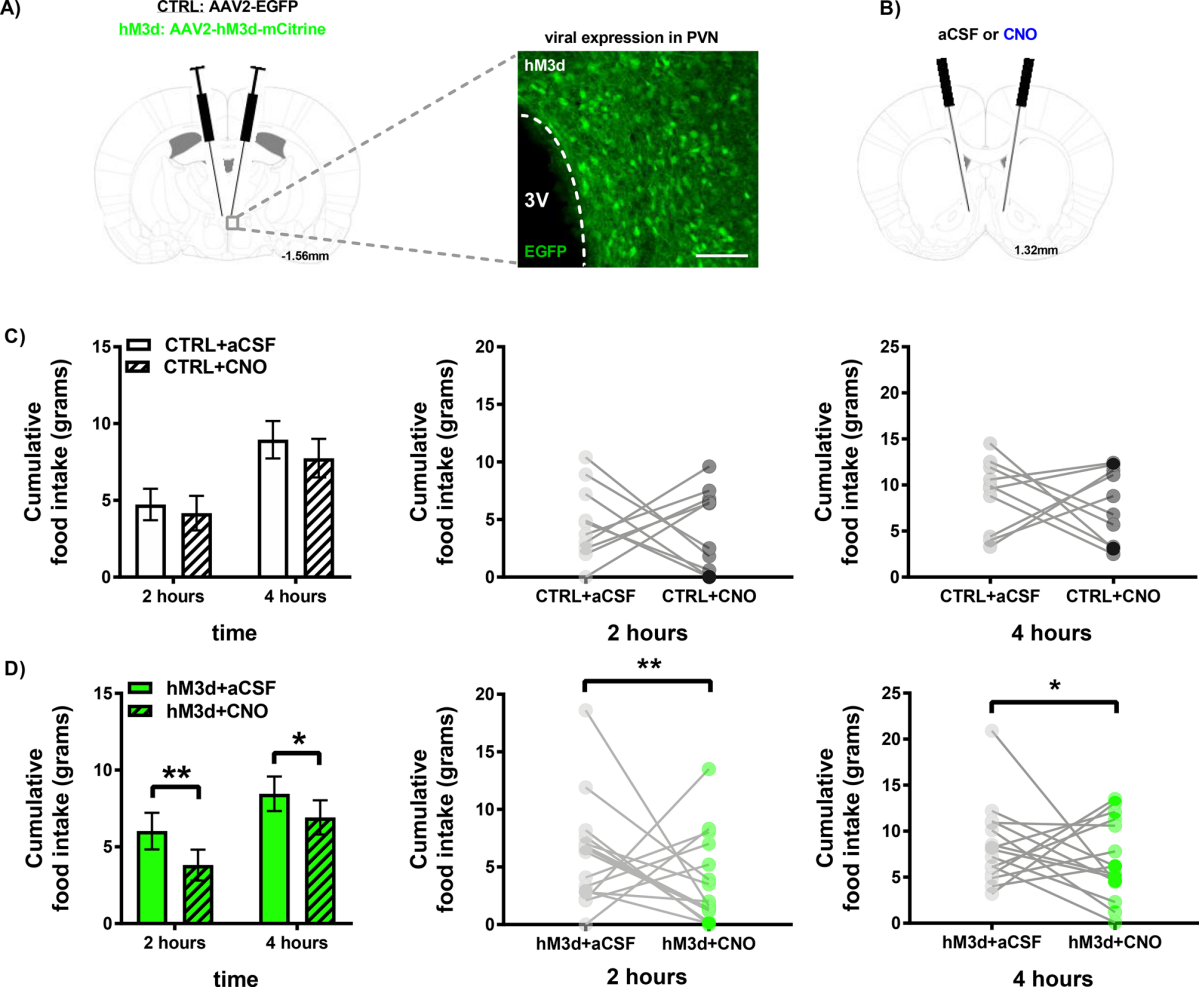
hM3d-induced stimulation of PVN → NAc decreases intake of HPF. (**A**) PVN hM3d viral targeting approach (left) and representative IHC image of hM3d viral expression in the PVN (right). (**B**) NAc guide cannula placement for aCSF or CNO administration. (**C**) Stimulation of PVN → NAc did not alter intake of HPF in CTRL + aCSF or CTRL + CNO rats at 2 h or 4 h post-administration (n = 11, *p* > 0.05 by 2-way RM-ANOVA). Cumulative food intake is also shown for each rat at 2 h (middle) and 4 h (right) post-aCSF or CNO administration. (**D**) Stimulation of PVN → NAc significantly decreased intake of HPF in hM3d + CNO rats compared to aCSF at 2 h post-administration (n = 15, **p < 0.01 by 2-way RM-ANOVA). Stimulation of PVN → NAc significantly decreased intake of HPF in hM3d + CNO rats compared to aCSF at 4 h post-administration, (n = 15, *p < 0.05 by 2-way RM-ANOVA). Cumulative intake of HPF is also shown for each rat at 2 h (middle) and 4 h (right) post-aCSF or CNO administration.

To determine whether stimulation of PVN → NAc and increased glutamatergic drive into the NAc affects feeding behavior, we utilized the same viral paradigm as performed with microdialysis experiments to express CTRL or hM3d in PVN → NAc neurons and quantified intake of highly palatable food (HPF) over a period of 4 h (Fig. 4). aCSF or CNO was micro-infused into the NAc of CTRL (CTRL + aCSF, CTRL + CNO) and hM3d (hM3d + aCSF, hM3d + CNO) rats immediately before rats were given ad libitum access to HPF (Fig. 4A). In this paradigm, each rat received aCSF and CNO on different test days to control for potential CNO-related effects on intake of HPF (Fig. 4B). No change in cumulative intake of HPF at 2 h or 4 h was observed in CTRL + aCSF or CTRL + CNO rats (Fig. 4C; n = 11; *p* > 0.05, no significance). However, at 2 h and 4 h, hM3d + CNO rats consumed significantly less HPF than hM3d + aCSF rats, resulting in a 37% and 18% reduction in HPF intake respectively (Fig. 4D; n = 15; 2 h: p > 0.01, significant; 4 h: p < 0.05, significant). Given that we observed a considerable decrease in intake of HPF in hM3d + CNO rats, our results demonstrate that glutamatergic drive into the NAc decreases intake of HPF.

## Discussion

Through viral tracing techniques, our results provide neuroanatomical evidence that the parvocellular compartment of the PVN directly interacts with the NAc. This finding corroborates an existing role for the parvocellular PVN in driving intake of HPF^20^. We observed co-localization of presynaptic VGLUT1 staining with PVN → NAc, indicating that PVN projections to the NAc are equipped for glutamate transport^21^. Because co-localization of PVN → NAc with VGLUT1 is not an indication of an exclusively glutamatergic pathway, we also characterized co-localization of PVN → NAc with other neurotransmitter markers. Our analysis revealed that PVN → NAc did not express markers associated with GABA, dopamine, or serotonin signaling. Taken together, our tracing studies support PVN → NAc as a crucial neural circuit that utilizes glutamate.

Selective pharmacogenetic stimulation of PVN → NAc neurons resulted in an increase in presynaptic extracellular glutamate in the NAc of hM3d rats. This finding corroborates our immunohistochemical results that PVN → NAc is predominantly comprised of glutamatergic neurons. We did not identify any changes in presynaptic extracellular GABA, suggesting that PVN → NAc neurons do not co-release GABA and glutamate. Our microdialysis design was limited by an inability to detect uncharged neurotransmitters (i.e. acetylcholine). We were also limited by the size of the microdialysis probe, which permitted collection of molecules less than 6 kDa, but not larger molecules such as neuropeptides (see “Methods”). Oxytocin is one neuropeptide released from PVN → NAc that has also been shown to decrease intake of HPF in humans and rodents^11,22^.

Our results indicate that glutamate released from PVN → NAc alters the excitability of neurons downstream from the circuit, as opposed to locally. We observed a significant increase in phospho-cFos expression in the NAc of hM3d + CNO rats only, suggesting that presynaptic glutamate release from PVN → NAc activates neurons in the NAc. In contrast, we observed minimal phospho-cFos expression in the PVN of CTRL + CNO and hM3d + CNO rats, which bolsters our approach in that only presynaptic DREADDs are stimulated by CNO administration. Further, we demonstrated that presynaptic glutamate release from PVN → NAc decreases intake of HPF, which aligns with previous work underscoring the significance of accumbal glutamate in decreasing both intake and motivation for food^9,23^. While feeding causes a decrease in extracellular glutamate release in the NAc during and after food intake^24^, pharmacological blockade of AMPA type glutamate receptors in the medial shell of the NAc elicits a robust increase in food intake while antagonism of the NMDA receptors in the same region did not affect food intake^9,25^. Here, we have defined a source of hypothalamic glutamatergic input to the NAc and demonstrated that stimulation of this input from the PVN into the NAc suppresses intake of HPF. However, one limitation is that we did not evaluate the effects of the PVN to NAc pathway on a less palatable diet, which will be evaluated in future studies.

While the PVN and the NAc are independently linked to feeding behaviors, we have elucidated PVN → NAc connectivity in regulating intake of HPF. We established the neuroanatomical properties of PVN → NAc and identified glutamate as the major neurotransmitter involved in PVN → NAc signaling. Through glutamatergic inputs to the NAc, the PVN decreases intake of HPF. Our identification of the role of hypothalamic neurons driving glutamate release in the NAc and regulating intake of HPF combined with existing literature on the robust effects of pharmacological manipulations of glutamate on feeding behaviors indicates that further investigation into the glutamate system as a potential therapeutic target for the treatment of maladaptive feeding behaviors is warranted.

## Methods

### Animals

Male Sprague Dawley rats (Harlan, Houston, TX) weighing 225–250 g were used in all experiments. Rats were housed individually in a temperature and humidity-controlled environment with a normal 12 h light/dark cycle (0600–1800). All experiments were conducted during the light phase. Rats had ad libitum access to food and water in their home cages. All experiments were conducted in accordance with the Guide for Use and Care of Laboratory Animals, and with approval from the Institutional Animal Use and Care Committee at the University of Texas Medical Branch and at the University of Florida.

### Viral tracers

For retrograde tracing of NAc inputs, rats (n = 3) received bilateral injection of a retrograde AAV6-GFP into the NAc (Fig. 1). To label terminals in the NAc, rats (n = 3) received bilateral injection of AAV2-CAMKIIa-EGFP (Fig. 2). All stereotaxic injections (Stoelting) were performed under 2–5% isoflurane anesthesia (VetEquip) aimed at the PVN or NAc shell at a 10 degree angle at the following coordinates: PVN: A/P − 1.8 mm; M/L + 2.0 mm; D/V − 8.2 mm from bregma; NAc: A/P + 1.4 mm; M/L + 2.0 mm; D/V − 6.8mm^26^. We targeted the NAcSh for its known involvement in reinforcement and goal-directed behavior. Rats recovered for 2 weeks post-surgery to ensure optimal viral expression.

### Immunohistochemistry and confocal microscopy

Rats were anesthetized with 5% isoflurane and perfused with 1× PBS for 5 min then 4% paraformaldehyde for 15 min. Whole brains were collected and immediately post-fixed in 4% PFA overnight. The following day, brains were moved to a solution of 1× PBS + 20% glycerol. Brain slices (40um) containing the PVN and/or the NAc were collected and stored in 1× PBS + 0.05% sodium azide until immunohistochemistry was performed. Briefly, slices were washed, unmasked, blocked, and incubated in primary antibody overnight. The next day, slices were washed in 1× PBS and incubated in secondary antibody for 2 h. Slices were washed in 1× PBS and mounted. Images were acquired using Leica True Confocal Scanner SPE in confocal mode and Leica Application Suite x software (Leica Microsystems).

The following primary antibodies were used: Anti-chicken GFP (1020, 1:200, Aves Labs; Anti-mouse vesicular glutamate transporter 1 (VGLUT1) (ab135311, 1:200, Synaptic Systems); Anti-mouse glutamate decarboxylase 67 (GAD_67_) (ab26116, 1:200, Abcam); Anti-mouse tyrosine hydroxylase (TH) (T2928, 1:500, Sigma); Anti-mouse tryptophan hydroxylase (TPH) (T0678, 1:500, Sigma); Anti-rabbit phospho-cFos (5348, 1:200, Cell Signaling) The following secondary antibodies were used: Donkey anti-chicken 488 (703-545-155, 1:200, Jackson ImmunoResearch); Donkey anti-mouse AlexaFluor 488 (A21202, 1:200, Invitrogen); Donkey anti-mouse AlexaFluor 555 (A31570, 1:200, Invitrogen); Donkey anti-rabbit AlexaFluor 568 (A10042 1:200, Invitrogen).

Neurons of the PVN are defined by their location within structurally established parvocellular or magnocellular regions^26^. These regions were superimposed on 10× tile scan images to outline a region of interest for quantification of GFP + cell bodies projecting to the NAc. Researcher quantification was confirmed by an objective quantification via Fiji Is Just Image J (FIJI, Image J). Briefly, a region of interest was applied to all images, then background subtracted. Image threshold was adjusted size and circularity parameters applied to quantify GFP + cell bodies.

### Viral and cannula surgeries for microdialysis

To determine the functional mechanism of PVN → NAc neurons, rats received bilateral injection of AAV2-CAMKIIa-EGFP (CTRL (plasmid 50,469, Addgene) or AAV2-CAMKII-HA-hM3d(G_q_)-IRES-mCitrine (hM3d) (plasmid 50,466, Addgene) into the PVN at A/P − 1.8 mm; M/L + 2.0 mm; D/V − 8.2 mm from bregma^26^. Unilateral guide cannula for CNO micro-infusion (C315GA/SPC26GA, PlasticsOne) cut 6 mm below the pedestal were implanted at a 20° outside angle at the following coordinates: A/P + 1.4 mm; M/L + 3.2 mm; D/V − 5.8 mm from bregma and a unilateral guide cannula for microdialysis probe placement was implanted at a 0 degree outside angle at the following coordinates: A/P + 1.4 mm; M/L − 0.18 mm; D/V − 5.3 mm from bregma^27^. Guide cannulae were secured with dental cement and stainless-steel screws. Microdialysis probes with 2 mm active length and 13,000 molecular weight cut off were constructed as previously described^28^. After calibration, probes were inserted in the guide cannula (see Fig. S1 for placement), connected to a dual channel swivel, and perfused with aCSF at 1 μL/min. The swivel was mounted atop a modified home cage lid so that animals could move freely and have access to food and water during the experiment.

### Microdialysis and capillary electrophoresis and laser-induce fluorescence (CE-LIF)

Neurotransmitter data was collected via microdialysis and quantified using with capillary electrophoresis with laser-induced fluorescence^28–30^. A standard curve (7 concentrations of glutamate, aspartate, GABA, glutamine, and glycine ranging from 0 to 20 μM) was generated using a microdialysis probe prior to beginning the experiment. The elution time of GABA and glutamate have been validated and characterized previously^29^. After calibration, the probe was implanted in a non-anesthetized and freely moving rat extending 2 mm beyond the guide cannula. A baseline for each animal was collected for 2 h prior to the experiment (average glutamate 6.20 µM, GABA 0.37 µM). For the experiment, 1uM CNO (6329, Tocris Biosciences) was administered via the guide cannula continuously at a rate of 0.5ul/min for 4 min, followed by 2 min before removal of internal cannula. Samples were quantified by online capillary electrophoresis every 20 s and averaged into 4 min bins. (Fig. 2a). Microdialysis probe placement and cannula placement were verified (Fig. S1) and all misses were eliminated prior to data analysis. Like GABA, we did not detect any changes in glycine, glutamine, or aspartate.

### Phospho-cFos and confocal microscopy

2 h prior to euthanasia, CTRL (CTRL + CNO: n = 3) and hM3d (hM3d + CNO: n = 3) rats received an intra-NAc microinfusion of 1uM CNO at a rate of 0.5ul/min for 4 min, followed by 2 min before removal of internal cannula. Rats were perfused with 1× PBS for 5 min followed by 4% paraformaldehyde for 15 min. Brains were removed and post-fixed for 24 h in 4% paraformaldehyde. Brains were stored in PBS glycerol sodium azide until slicing. Immunohistochemistry was conducted as previously described^17,18,27,31^. Phospho-cFos was immuno-enhanced using anti-rabbit phospho-cFos (Ser 32) (Cell Signaling, 5348), then visualized in both the PVN and the NAc. Tile scans were acquired at 10× using Leica True Confocal Scanner SPE in confocal mode and Leica Application Suite x software (Leica Microsystems). Images were analyzed for phospho-cFos expression using FIJI (Image J). A region of interest was created in FIJI and applied to all images. The following parameters were applied to all images for analysis: rolling ball radius: 20.0, threshold: 0–20, particle size: 50-infinity (pixel units), circularity: 0.00–1.00.

### Viral and cannula surgeries for HPF intake study

To determine the behavioral output of PVN → NAc neurons, rats received bilateral injection of AAV2-CAMKIIa-EGFP (CTRL (plasmid 50469, Addgene) or AAV2-CAMKII-HA-hM3d(G_q_)-IRES-mCitrine (hM3d) (plasmid 50466, Addgene) into the PVN at A/P − 1.8 mm, M/L + 2.0 mm, D/V − 8.2 mm from bregma^26^. Bilateral guide cannulae (PlasticsOne; 26 gauge, cut 6 mm below pedestal) were implanted at 10° outside angle at the following coordinates: A/P + 1.4 mm; M/L + 1.8 mm; D/V − 5.8 mm from bregma and secured with dental cement and stainless-steel screws. After surgery, animals recovered for 2 weeks to allow for optimal virus expression. Animals were habituated to cannula manipulation starting 1 week prior to CNO/aCSF administration. Virus and cannula placement were verified and all misses were eliminated prior to data analysis.

### HPF intake study

On experimental day 1, rats (n = 26 total; CTRL: n = 11; hM3d: n = 15) received intra-NAc micro-infusion of aCSF or 1uM CNO via internal cannulae (PlasticsOne; 33 gauge, extending 2 mm beyond the end of the guide cannulae) immediately prior to onset of the dark cycle (1800). A total of 2ul of aCSF or 1uM CNO was infused at a rate of 0.5ul/min for 4 min, followed by a 2 min wait period before removal of the injector. Food hoppers containing 45% high fat chow (D12451, Research Diets) were placed in home cages and rats were given ad libitum access for 4 h. Food hopper weights were recorded at 2 h and 4 h post micro-infusion. This same paradigm was used to assess food intake on experimental day 2, where rats received aCSF (CNO on day 1) or 1uM CNO (aCSF on day 1) so that each rat served as its own control.

### Locomotor activity

Locomotor activity was assessed in rats included in the food intake study (n = 26 total; CTRL: n = 11; hM3d: n = 15). aCSF and 1 µM CNO was administered immediately prior to a 30-min locomotor session during which horizontal beam breaks were quantified (Fig. S2a).

### Statistical analysis

Data were analyzed using Prism 6.0 (GraphPad) and FIJI (ImageJ). For animal surgeries, CTRL and hM3d viruses were interleaved to avoid any effect of surgeon. For behavioral testing, each animal received aCSF and CNO on separate test days to control for aCSF or CNO effects.
